# Artichoke (*Cynara scolymus L.*) water extract alleviates palmitate-induced insulin resistance in HepG2 hepatocytes via the activation of IRS1/PI3K/AKT/FoxO1 and GSK-3β signaling pathway

**DOI:** 10.1186/s12906-023-04275-3

**Published:** 2023-12-15

**Authors:** Aihua Deng, Yun Wang, Kerui Huang, Peng Xie, Ping Mo, Fengying Liu, Jun Chen, Kaiyi Chen, Yun Wang, Bing Xiao

**Affiliations:** 1 Key Laboratory of Agricultural Products Processing and Food Safety in Hunan Higher Education; Science and Technology Innovation Team for Efficient Agricultural Production and Deep Processing at General University in Hunan Province; Human Provincial Engineering Research Center for Fresh Wet Rice Noodels; College of Life and Environmental Science, Hunan University of Arts and Science, Changde, 415000 China; 2 Sanjin Group Hunan Sanjin Pharmaceutical Co., Ltd, Changde, 415000 China; 3 National Clinical Research Center for Hematologic Diseases, Jiangsu Institute of Hematology, the First Affiliated Hospital of Soochow University, Soochow University, Suzhou, P. R. China; 4 Institute for Development and Regenerative Cardiovascular Medicine, Xinhua Hospital, Shanghai Jiao Tong University School of Medicine, Shanghai, 200092 China

**Keywords:** Artichoke, Insulin resistance, HepG2 cells, Gluconeogenesis, Glycogen synthesis

## Abstract

**Background:**

Artichoke (*Cynara scolymus L.*) is a typical element of a traditional Mediterranean diet and has potential health advantages for insulin resistance (IR) and type 2 diabetes mellitus (T2DM). This study aims to evaluate the effect and underlying mechanism of artichoke water extract (AWE) on palmitate (PA)-induced IR in human hepatocellular carcinoma (HepG2) cells.

**Methods:**

The effect of AWE on cell viability was determined using CCK8 assay. Cellular glucose uptake, glucose consumption, glucose production, and glycogen content were assessed after AWE treatment. The gene expression and protein levels were examined by real-time polymerase chain reaction (qRT-PCR) and western blotting.

**Results:**

The results showed that AWE dose-dependently increased cell viability in IR HepG2 cells (*P* < 0.01). AWE treatment significantly promoted glucose uptake and consumption, decreased glucose production, and increased the cellular glycogen content in IR HepG2 cells (*P* < 0.01). Mechanistically, AWE elevated the phosphorylation and total protein levels of major insulin signaling molecules in IR HepG2 cells, which resulted in a decrease in the expression of phosphoenolpyruvate carboxykinase (PEPCK) and glucose-6-phosphatase (G6Pase) and the inhibition of glycogen synthase (GS) phosphorylation in IR HepG2 cells. Furthermore, the protective effect of AWE on IR HepG2 cells might be ascribed to the inhibition of the endoplasmic reticulum (ER) stress.

**Conclusion:**

We conclude that AWE may improve glucose metabolism by regulating IRS1/PI3K/AKT/FoxO1 and GSK-3β signaling associated with the inhibition of ER stress in IR HepG2 cells induced by PA.

**Supplementary Information:**

The online version contains supplementary material available at 10.1186/s12906-023-04275-3.

## Introduction

Type 2 diabetes mellitus (T2DM) is a metabolic disease characterized by chronic hyperglycemia due to impaired insulin secretion and insulin resistance (IR) [1]. IR, a defective response to insulin stimulation of target cells such as hepatocytes, skeletal muscle cells, and adipocytes, plays a crucial role in the development of several metabolic diseases including T2DM, obesity, metabolic dysfunction-associated fatty liver disease, and dyslipidemia [2]. The pathogenic mechanism of IR is complex and still not fully understood, and evidence is accumulating that high concentrations of plasma-free fatty acids (FFAs) are closely correlated with the increased risk of developing IR and T2DM [3]. Numerous studies have shown that palmitic acid (PA), the most abundant saturated FFA in circulation, triggers excessive reactive oxygen species production due to disrupting mitochondrial function and inducing oxidative stress, which has been implicated in the pathogenesis of IR associated with the endoplasmic reticulum (ER) stress [4]. In addition, ER stress and the subsequent unfolded protein response (UPR) also have been implicated in the impaired insulin signaling in hepatocytes caused by PA [5–7].

Insulin-stimulated activation of the IRS1/PI3K/Akt pathway plays a critical role in regulating the hepatic expression of glucose-metabolizing genes involved in glycolysis, gluconeogenesis, and glycogenesis [8, 9]. On the one hand, Akt activation induces a decrease in hepatic gluconeogenesis by downregulating phosphoenolpyruvate carboxykinase (PCPCK) and glucose-6-phosphatase (G6Pase) by promoting phosphorylation of forkhead box transcription factor O1 (FoxO1), resulting in a cytoplasmic translocation of FoxO1 and repression of FoxO1-mediated transactivation [10]; On the other hand, activation of Akt leads to increased hepatic glucose uptake and increased hepatic glycogen synthesis (glycogenesis) by upregulating expression of hepatic glucose transporter 2 (GLUT2) [11] and by reducing glycogen synthase (GS) phosphorylation, which is directly regulated by glycogen synthase kinase 3 beta (GSK3β) [12]. Therefore, targeting hepatic glucose metabolism mediated by IRS1/PI3K/Akt signaling is an accepted strategy for the prevention and treatment of IR and T2DM.

Lifestyle and pharmacologic interventions are considered available strategies to alleviate T2DM [13], however, no specific drugs have yet been approved to treat IR even diabetes medications (such as metformin and thiazolidinediones, or thiazolidinediones) are insulin sensitizers that lower blood glucose partly by reducing IR. Recently, the use of natural functional dietary supplements appears to be an attractive strategy to develop safe and effective drugs to treat IR. For example, some edible plants and their active compounds have been shown to improve hepatic glucose and lipid metabolism and alleviate IR and T2DM [14–16]. Artichoke (*Cynara scolymus L.*), a member of the Asteraceae family, is commonly used as a healthy food and as a popular traditional herbal remedy to protect the liver function [17, 18]. The active compounds identified in artichokes, including chlorogenic acid, cynarin, flavonoids and their derivatives, have attracted considerable attention for their multiple pharmacological functions, including antioxidant stress, hypolipidemic, antidiabetic, anti-inflammatory and anticancer effects [19–21]. Our previous study showed that artichoke water extract (AWE) has an anti-IR effect in high-fat diet-induced nonalcoholic fatty liver disease in rats [22]. In this study, to further investigate the molecular mechanism of the anti-IR effect of AWE, we used palmitate (PA)-induced IR in human HepG2 hepatocytes to study the anti-IR effect of AWE and the underlying mechanisms.

## Materials and methods

### Materials

Artichoke (*Cynara scolymus L.*) has been checked with “World Flora Online” (www.worldfloraonline.ogr), and was certificated by Hunan Crop Variety Examination and Approval Committee. The harvested plants were identified in the botany laboratory of hunan college of arts and sciences, changed, by Professor Youlin Peng and a voucher specimen [CXJ24] was deposited at the herbarium of the laboratory of the center for biological and agricultural resources of Hunan College of Arts and Sciences. AWE (containing 1.2% chlorogenic acid, 4.8% cynarin) was supplied by Huimei Agricultural Science and Technology Co. Ltd. (Hunan, China) as described in our previous study [22]. Sterile bovine serum albumin (BSA) coupled sodium salt of PA solution (2.5% BSA) and control solvent (containing 2.5% fatty acid-free BSA) was ordered from Shanghai Siduorui Biotechnology Service Co., Ltd (Shanghai, China). CCK8 was purchased from Yeasen Biotech Co., Ltd (Shanghai, China). Dulbecco’s Modified Eagle Medium (DMEM) was acquired from BasalMedia Technologies Co., Ltd (Shanghai, China). Fetal bovine serum (FBS) was purchased from GIBCO Co., Ltd (Shanghai, China). 2-Deoxy-2-[(7-nitro-2,1,3-benzoxadiazol-4-yl)amino]-D-glucose (2-NBDG) and metformin were purchased from Shanghai Yuanye Biotechnology Co., Ltd (Shanghai, China). Primary antibodies against IRS1, p-IRS1(Tyr612), Akt, p-Akt (Ser473), p-GSK3β (Ser9), p-FoxO1 (Ser256), GS, p-GS (Ser641), ATF6, GRP78 and CHOP were ordered at Cell Signaling Technology (Boston, USA). PI3K p100α, GSK3β, GLUT2, PEPCK, G6pase, FoxO1 and GAPDH were purchased from Santa Cruz Biotechnology (Santa Cruz, USA).

### Methods

#### PA-induced IR in HepG2 cells

The HepG2 cells were purchased from the American Type Culture Collection (ATCC) and incubated in DMEM (Thermo Fisher Scientific) with 10% FBS at 37 °C with 5% CO_2_. To establish a PA-induced IR model in HepG2 cells, the cells were incubated with the indicated concentrations of PA (sodium salt of PA) for 24 h as previously described [23], and the control group treated with control solvent (containing 2.5% fatty acid-free BSA). Six replicates were performed for each group and repeated three times.

### Cell viability assay

The effect of AWE on the viability of PA-induced HepG2 cells was measured using the Cell Counting Kit-8 (CCK-8) assay. In brief, cells were seeded in 96-well plates for 24 h and then exposed to PA at different AWE concentrations for an additional 24 h. After treatment, CCK8 solution was added and incubated for 1 h. The optical density (OD) value was then measured at 450 nm. The cell viability value in the control group was set as 100%. Six replications *per* group were performed and repeated three times.

### Glucose consumption assay

The glucose consumption test was performed according to the previous publication [16] with minor modifications. Briefly, HepG2 cells were seeded in 96-well plates, leaving 6 wells as a blank control. After 24 h of incubation, cells were exposed to PA with or without AWE or metformin (100 µM) for 24 h. After treatment, the culture medium was removed and the cells were incubated with complete DMEM containing insulin (100 nM) for 30 min. Subsequently, the cells were washed twice with PBS and the culture medium was replaced with DMEM containing 11.1 mmol/L glucose supplemented with 0.5% FBS. After 24 h, the culture medium was collected and the glucose consumption was calculated by the glucose concentrations of blank wells minus the glucose concentrations in plated wells using the glucose kit (Nanjing Jian cheng, Nanjing, China). The CCK8 assay was used to adjust for the glucose consumption. Experiments were performed in six replicates and repeated three times.

### Glucose uptake assay

The glucose uptake test was performed as in the previous report with minor modifications [24]. Briefly, the cells were cultured for 24 h in 24-well plates. Cells were then exposed to PA with or without AWE or metformin (100 µM) for 24 h. The cells were then exposed to insulin (100 nM) for 30 min and further incubated with 2-NBDG (300 µM) for 45 min. The images were then recorded using a fluorescence microscope (Zeiss). Fluorescence intensity was analyzed using Image J software. Fluorescence intensity was normalized with cell number. Six images were calculated *per* group and this experiment was repeated three times.

### Glycogen content assay

The glycogen content test was performed as previously described with some modifications [16]. HepG2 cells were cultured for 24 h in 6-well plates. Subsequently, the cells were exposed to PA for 24 h in the presence or absence of AWE or metformin (100 µM). After the treatments, the cells were washed twice with PBS and incubated with fresh culture medium containing insulin (100 nM) for 30 min. Then, the glycogen content of the cells was evaluated with a glycogen assay kit (Solarbio, Beijing, China). Glycogen content was normalized with cell count. Experiments were performed in three replicates and repeated three times.

### Glucose production assay

The glucose production test was performed according to the published procedure [16]. Briefly, the cells were seeded into 24-well plates and incubated for 24 h. Cells were then exposed to PA with or without AWE or metformin (100 µM) for 24 h. After the treatments, the cells were treated with insulin (100 nM) for 30 min, and washed three times with PBS. Cells were then treated with insulin (100 nM) in 300 µL of glucose production medium (glucose- and phenol red-free DMEM containing gluconeogenic substrates, including 20 mM sodium lactate and 2 mM sodium pyruvate) for 16 h. Then the glucose concentration was measured and normalized with the total protein content. Experiments were performed in six replicates *per* group and repeated three times.

### Western blot analysis

The western blot was performed as previously described with minor modifications [25]. After treatment, HepG2 cells were washed twice with cold PBS and then lysed with cold RIPA (RadioImmunoprecipitation Assay) protein extraction buffer containing 1% protease and phosphatase inhibitors (Beyotime Biotechnology, Beijing, China) for 25 min on ice. Twenty milligrams of supernatant protein from each sample was diluted with sodium dodecyl sulfate (SDS) sample buffer and heated at 95 °C for 10 min. The protein sample was electrophoresed on a 10% SDS–polyacrylamide gel and transferred to a PVDF membrane, which was then blocked with 5% BSA in TBS-T for 2 h at room temperature. While the size of the protein was already known, the blots were cut prior to hybridization with primary antibodies. The blocked membrane was incubated with primary antibodies overnight at 4 °C (1:1,000 dilution). Appropriate HRP-labeled secondary antibodies were selected to incubate with the membrane for 2 h at room temperature. Subsequently, the blot bands were visualized with an ECL reagent and imaged with the ChemiDoc MP Imaging System (Bio-Rad Laboratories (Shanghai) Co., Ltd., Shanghai, China) and the blots were analyzed with Image J software. The experiment was repeated three times.

### Real-time quantitative polymerase chain reaction (qRT-PCR)

qRT-PCR was performed as previously reported [25]. After treatment, HepG2 cells were washed twice with cold PBS and cellular total ribonucleic acid (RNA) was extracted with Trizol reagent (Invitrogen, USA). The cDNA was obtained using the Prime Script™ RT reagent kit (Takara, Japan) according to the manufacturer’s protocol. Subsequently, qRT-PCR was performed using the SYBR Green™ Premix Ex Taq™ II Kit (Takara Biotechnology, Dalian, China) with a Step One Plus Real Time PCR System (Applied Biosystems, CA, USA). Table 1 shows the primer sequences of various genes. Data were analyzed by relative quantification using the ^ΔΔ^Ct method and normalized to endogenous control GAPDH. Experiments were performed in six replicates *per* group and repeated three times.

**Table 1:** The primer sequences for the real-time PCR

Gene	Forward (5' > 3')	Reverse (5' > 3')
*IRS1*	CCCAGGACCCGCATTCAAA	GGCGGTAGATACCAATCAGGT
*PI3K p100α*	CCCAGGTGGAATGAATGGCT	AGCACCCTTTCGGCCTTTAA
*AKT*	AGCGACGTGGCTATTGTGAAG	GCCATCATTCTTGAGGAGGAAGT
*GLUT2*	GCCTGGTTCCTATGTATATCGGT	GCCACAGATCATAATTGCCCAAG
*PEPCK*	GGCTGAGAATACTGCCACACT	ACCGTCTTGCTCTCTACTCGT
*G6Pase*	ACTGGCTCAACCTCGTCTTTA	CGGAAGTGTTGCTGTAGTAGTCA
*ATF6α*	AGCAGCACCCAAGACTCAAAC	GCATAAGCGTTGGTACTGTCTGA
*GRP78*	CATCACGCCGTCCTATGTCG	CGTCAAAGACCGTGTTCTCG
*CHOP*	TTCTCTGGCTTGGCTGACTG	TCCTCCTCTTCCTCCTGAGC
*GAPDH*	TCCAAAATCAAGTGGGGCGA	TGATGACCCTTTTGGCTCCC

*IRS1* Insulin receptor substrate 1, *PI3K p100α* Phosphatidylinositol-4,5-bisphosphate 3-kinase catalytic subunit alpha, *Akt* Serine/threonine kinase 1, *GLUT2* Glucose Transporter 2, *PEPCK* Phosphoenolpyruvate carboxykinase 2, *G6Pase* Glucose-6-phosphatase catalytic subunit 1, *ATF6α* Activating transcription factor 6 alpha, *GRP78* G protein-coupled receptor 78, *CHOP* C/EBP homologous protein, *GAPDH* Glyceraldehyde-3-phosphate dehydrogenase

### Statistical analysis

Statistical analysis was performed using GraphPad Prism version 5.0 (GraphPad software, San Diego, CA, USA). Data are presented as mean ± standard deviation (SD). Statistical significance was determined by one-way ANOVA followed by Dunnett’s multiple comparisons. *P* < 0.05 was considered as statistically significant.

## Results

### Effect of AWE on the HepG2 cell viability

**Fig. 1:**
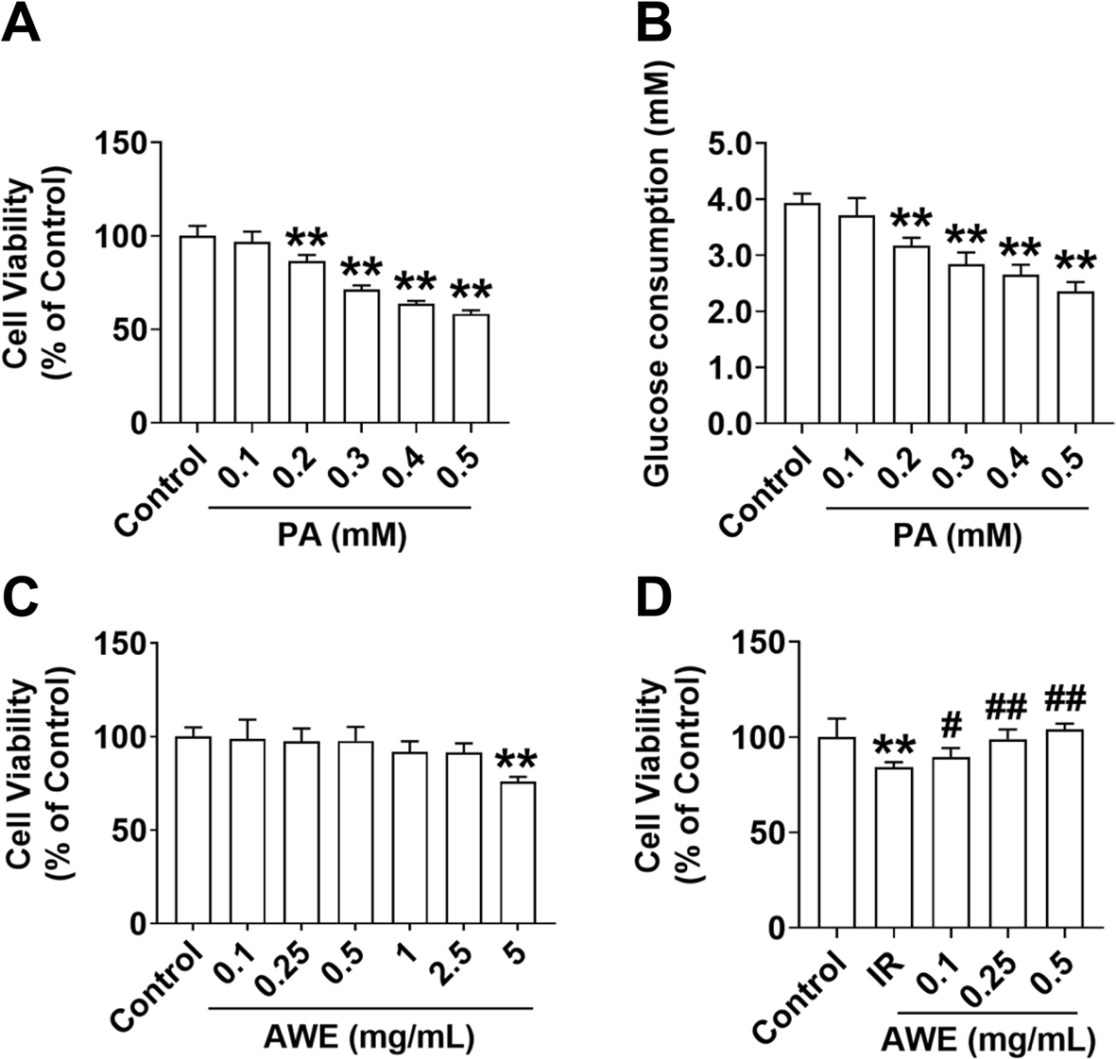
AWE increased the cell viability in IR HepG2 cells. **A** CCK8 assay was performed to examine the viability of HepG2 cells exposed to indicated concentrations of PA for 24 h. **B** Glucose consumption was measured in HepG2 cells exposed to indicated concentrations of PA for 24 h. **C** CCK8 assay was performed to examine the viability of HepG2 cells exposed to indicated concentrations of AWE for 24 h. **D** The effect of AWE on the viability of IR HepG2 cells was evaluated using the CCK8 assay. The IR group was treated with 0.2 mM PA for 24 h, and the control group was treated with the same volume of solution control (containing 2.5% fatty acid-free BSA). The data are expressed as mean ± SD. *n* = 6 *per* group. ^**^P < 0.01 *vs.* Control group. ^#^*P* < 0.05, ^##^*P* < 0.01 *vs.* IR group. The experiment was repeated three times

PA is typically used to induce IR in HepG2 cells with reduced glucose consumption in vitro [23]. The data showed that 24 h PA treatment dose-dependently reduced cell viability (*P* < 0.01) (Fig. 1A) and glucose consumption (*P* < 0.01) (Fig. 1B) in HepG2 cells. Considering the strong cytotoxic effect of high concentrations (0.3 to 0.5 mM) of PA (Fig. 1A), 0.2 mM PA could decrease glucose consumption in HepG2 cells although with little cytotoxicity. Thus, 0.2 mM PA was chosen for the establishment of the IR model in HepG2 cells in the following experiments.

To assess the toxicity of AWE to HepG2 cells, the reported AWE concentrations were evaluated against cell viability using the CCK8 assay. After 24 h of AWE treatment at 0.1 mg/mL to 2.5 mg/mL, no significant difference in cell viability was observed compared to the control group (Fig. 1C), suggesting that AWE may have a wide range of safety concentration (0.1 to 2.5 mg/mL). However, HepG2 cell viability was significantly reduced when treated with 5 mg/mL AWE (*P* < 0.01) (Fig. 1C). We next examined the effect of AWE on the PA-induced decreased cell viability of IR-HepG2 cells. As shown in Fig. 1D, the viability of HepG2 cells was significantly decreased in the IR group compared to the control group (*P* < 0.01), while AWE treatment at 0.1 to 0.5 mg/mL dose-dependently increased the viability of HepG2 cells in the presence of PA (*P* < 0.01).

### Effect of AWE on glucose metabolism

**Fig. 2:**
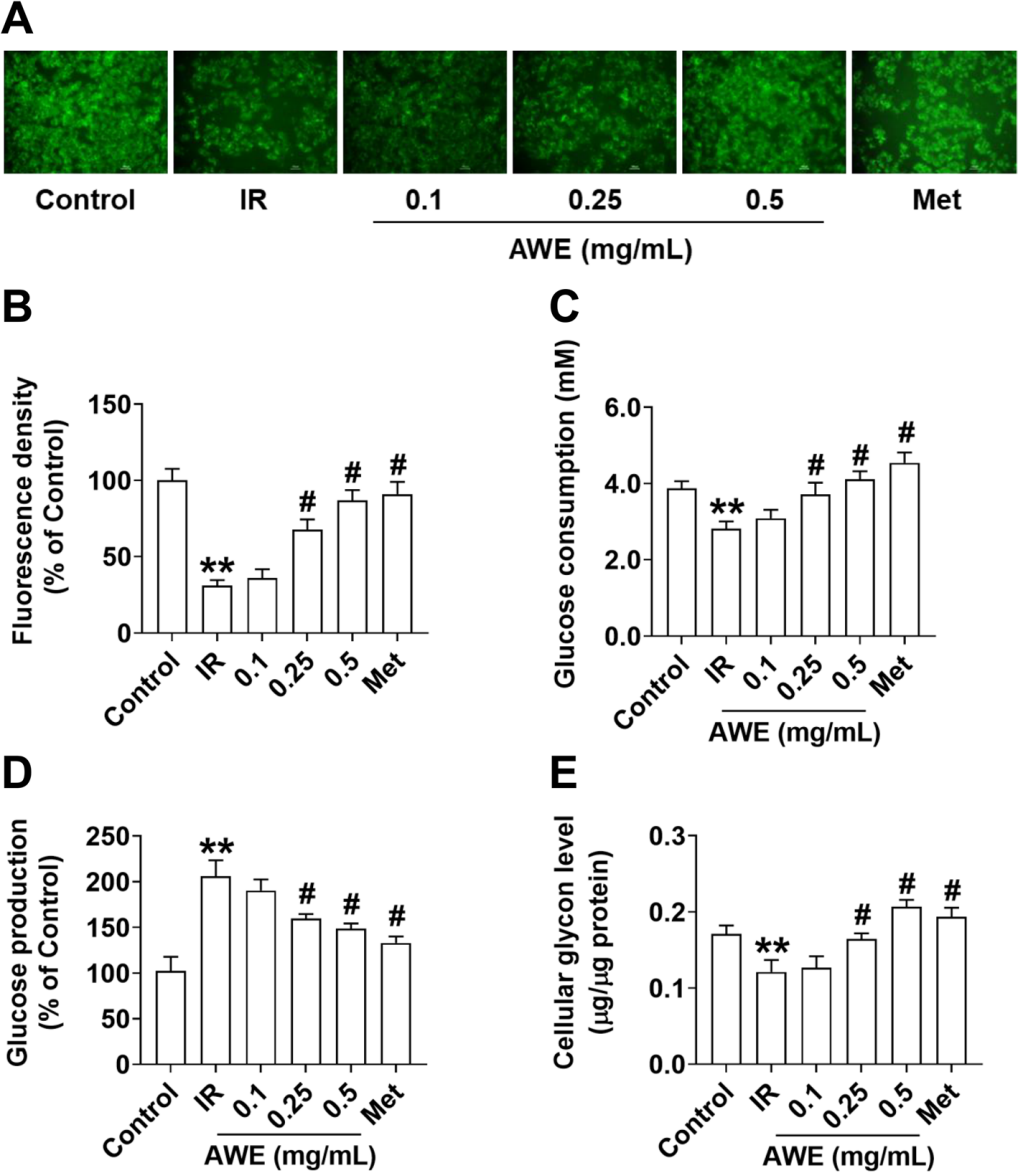
AWE improved glucose metabolism in IR HepG2 cells. HepG2 cells were exposed to 0.2 mM PA with or without indicated concentrations (0.1, 0.25, and 0.5 mg/mL) of AWE or 100 µM metformin (Met) for 24 h and then treated with 100 nM insulin for 30 min. **A** The glucose uptake was detected by 2-NDBG assay. **B** The fluorescence intensity of these fluorescent images was analyzed using Image J software. **C**-**E** Glucose consumption (**C**), glucose production (**D**) and the cellular glycogen content (**E**) were measured. The data are expressed as mean ± SD. *n* = 6 *per* group. ^**^P < 0.01 *vs.* Control group. ^#^*P* < 0.05 *vs.* IR group. The experiment was repeated three times

Compared to the control group, glucose uptake was significantly reduced in the IR group (*P* < 0.01) (Fig. 2A and B). However, similar to the effect of metformin (positive control group), AWE at 0.25 and 0.5 mg/mL significantly improved the impairment of glucose uptake in PA-treated HepG2 cells (*P* < 0.01) (Fig. 2A and B). Furthermore, similar to metformin, AWE treatment increased glucose consumption in a dose-dependent manner compared to the IR group (*P* < 0.01) (Fig. 2C). As shown in Fig. 2D, a significant increase in cellular glucose production was observed in the IR group compared to the control group (*P* < 0.01). As expected, there was a significant decrease in cellular glucose production in the metformin-treated group (Met group) compared to the IR group (*P* < 0.01) (Fig. 2D). Similar to the effect of metformin, AWE dose-dependently suppressed cellular glucose production in IR HepG2 cells (*P* < 0.01) (Fig. 2D). There was a significant decrease in cellular glycogen content in the IR group compared to the control group (*P* < 0.01) (Fig. 2E). However, cellular glycogen content was increased in the Met group compared to the IR group (*P* < 0.01) (Fig. 2E). Similar to metformin, AWE dose-dependently increased cellular glycogen content (*P* < 0.01) (Fig. 2E). These results suggest that AWE improves glucose metabolism in IR-HepG2 cells by inhibiting gluconeogenesis and increasing glycogen synthesis.

### Effect of AWE on the insulin-mediated IRS1/PI3K/Akt signal

**Fig. 3:**
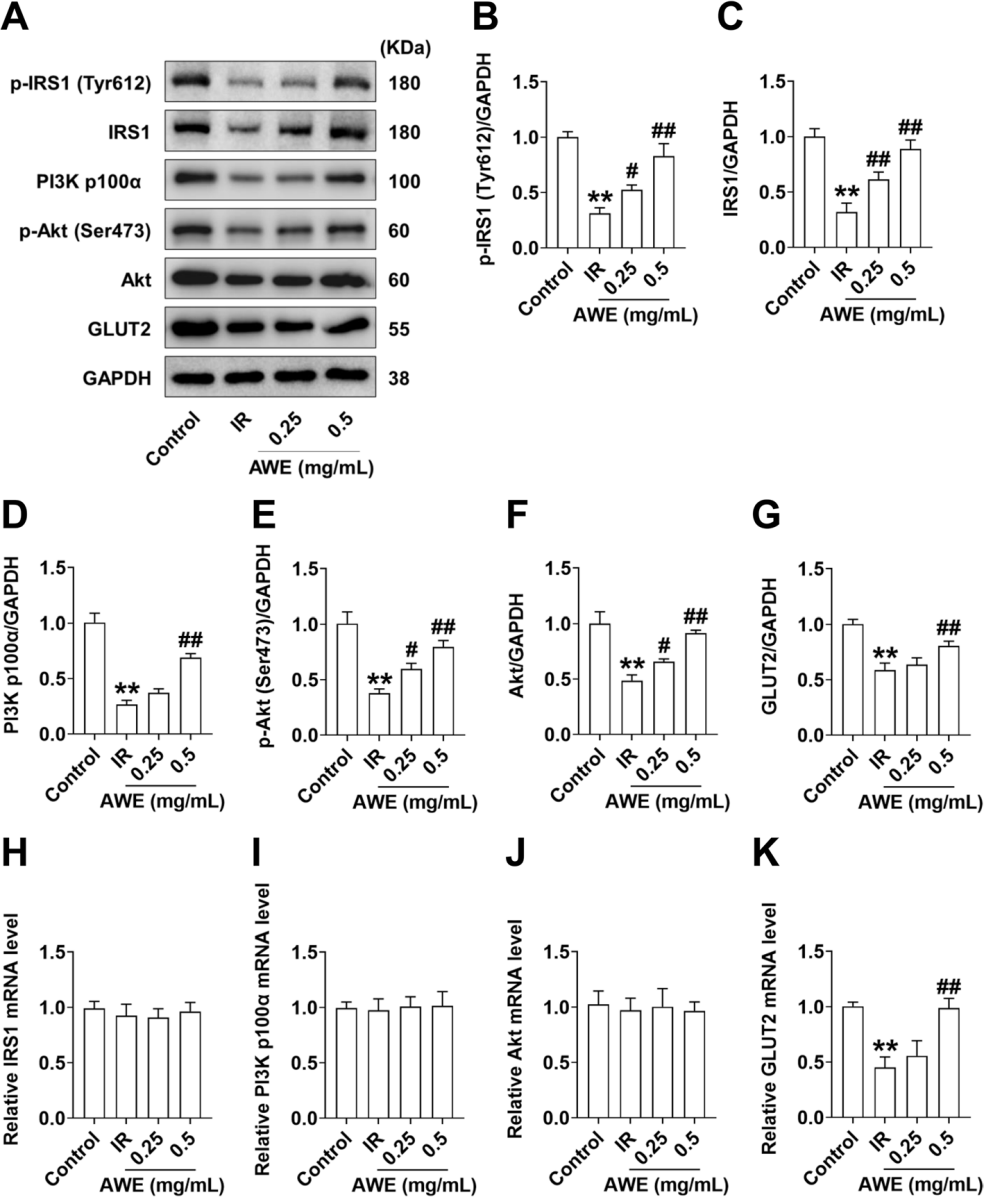
AWE improved the IRS1/PI3K/Akt signaling pathway in IR HepG2 cells. HepG2 cells were exposed to 0.2 mM PA in the absence or presence of 0.25 mg/mL and 0.5 mg/mL AWE for 24 h and then incubated with 100 nM insulin for 30 min. **A** Representative Western blot images of p-IRS1(Tyr 612), IRS1, PI3K p100α, p-Akt (Ser 473), Akt, GLUT2, and GAPDH. **B**-**G** Quantitative analysis of p-IRS1(Tyr612) (**B**), IRS1 (**C**), PI3K p100α (**D**), p-Akt (Ser473) (**E**), Akt (**F**), and GLUT2 (**G**). GAPDH is used as a loading control for protein normalization. **H**–**K** Relative mRNA expression of IRS1 (**H**), PI3K p100α (**I**), Akt (**J**), and GLUT2 (**K**) was quantified by qRT-PCR. mRNA levels of target genes were normalized to GAPDH. The data are expressed as mean ± SD. *n* = (3—6) *per* group. ^**^P < 0.01 *vs.* Control group. ^#^*P* < 0.05, ^##^*P* < 0.01 *vs.* IR group. The experiment was repeated three times

To investigate whether AWE has a protective effect on PA-induced defects in insulin signaling molecules, the expression and phosphorylation levels of key protein molecules in the insulin signaling pathway were analyzed with Western blot. As shown in Fig. 3A-F, there was a significant decrease in protein expression levels of p-IRS1(Tyr612) (Fig. 3B), IRS1 (Fig. 3C), PI3K p100α (Fig. 3D), p-Akt (Ser473) (Fig. 3E) and Akt (Fig. 3F) in PA-induced IR-HepG2 cells. In contrast, AWE treatment increased the phosphorylation and total protein levels of these molecules in a concentration-dependent manner (Fig. 3A-F) without affecting the mRNA expression levels of these genes, including IRS1 (Fig. 3H), PI3Kp100α (Fig. 3I), and Akt (Fig. 3J), suggesting that the inhibitory effect of AWE on PA-induced down-regulation of key molecules involved in IRS1/PI3K/Akt signaling may be related to post-translational modifications but not to transcriptional regulation. In addition, AWE treatment also improved PA-induced loss of GLUT2 protein and mRNA levels (Fig. 3A, G, and K). These results suggest that AWE activates the IRS1/PI3K/Akt signaling in IR-HepG2 cells.

### Effect of AWE on the expression of gluconeogenesis-related genes

**Fig. 4:**
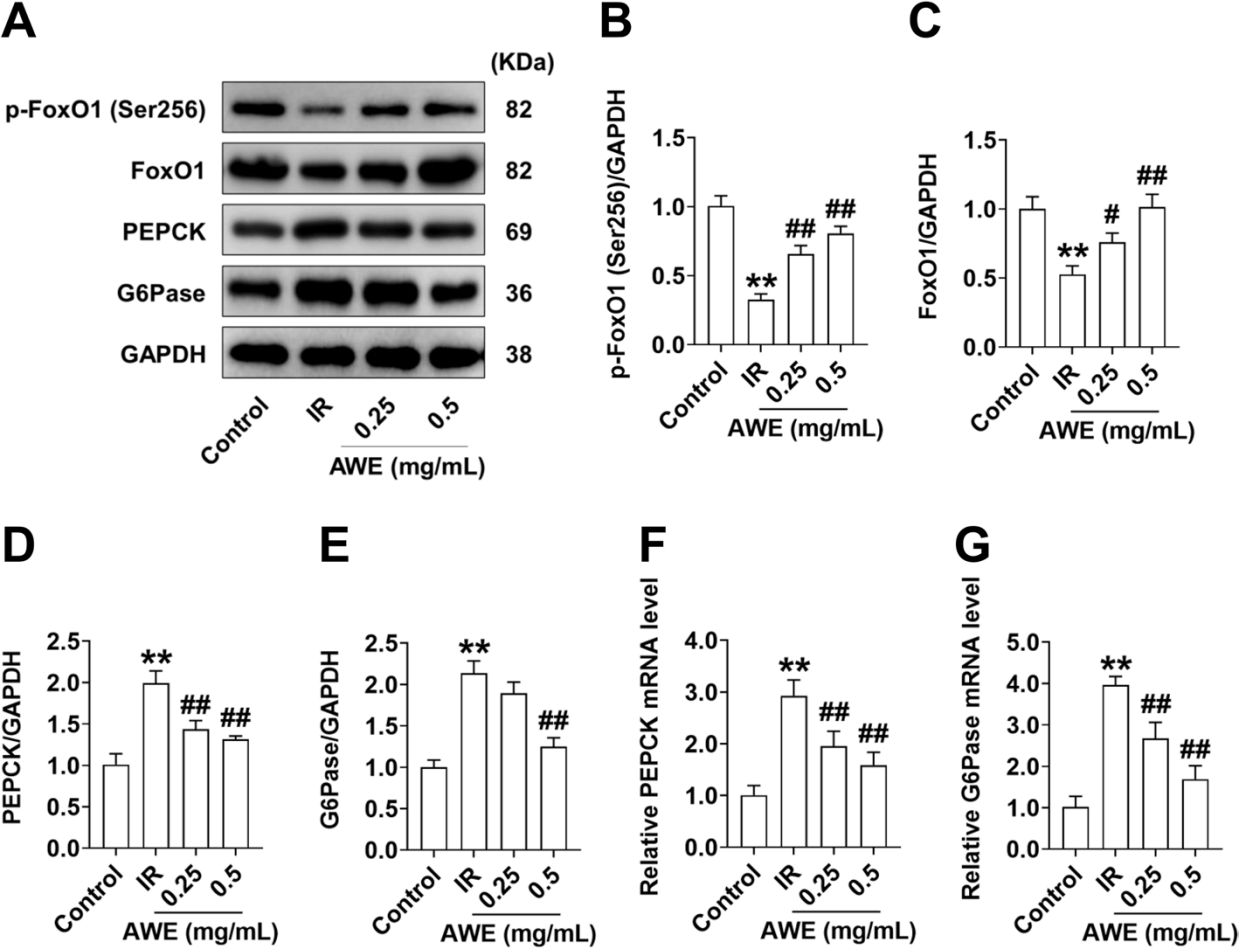
AWE inhibited the expression of key genes involved in gluconeogenesis in IR HepG2 cells. HepG2 cells were exposed to 0.2 mM PA in the absence or presence of 0.25 mg/mL and 0.5 mg/mL AWE for 24 h and then incubated with 100 nM insulin for 30 min. **A** Representative Western blot images of p-FoxO1(Ser256), FoxO1, PEPCK, G6Pase, and GAPDH. **B**-**E** Quantitative analysis of p-FoxO1(Ser256) (**B**), FoxO1 (**C**), PEPCK (**D**), G6Pase (**E**). GAPDH is used as a loading control for protein normalization. **F**&**G** Relative mRNA expression of PEPCK (**F**) and G6Pase (**G**) was quantified by qRT-PCR. mRNA levels of target genes were normalized to GAPDH. The data are expressed as mean ± SD. *n* = (3—6) *per* group. ^**^P < 0.01 *vs.* Control group. ^#^*P* < 0.05, ^##^*P* < 0.01 *vs.* IR group. The experiment was repeated three times

We next examined the effect of AWE on the expression of downstream signals in the Akt pathway associated with gluconeogenesis. The results showed that p-FoxO1(Ser256) (Fig. 4A, B) and FoxO1 (Fig. 4A, C) protein levels were down-regulated in IR-HepG2 cells (*P* < 0.01). Accordingly, a significant increase in mRNA and protein levels of PEPCK and G6Pase was observed in IR-HepG2 cells (*P* < 0.01) (Fig. 4A, D-G), suggesting that gluconeogenesis is increased in IR-HepG2 cells consistent with the result described above (Fig. 2D). However, AWE treatment dose-dependently increased p-FoxO1(Ser256) and total FoxO1 protein expression and downregulated mRNA and protein levels of PEPCK and G6Pase (*P* < 0.01) (Fig. 4A, F, and G). These results suggest that AWE may alleviate IR in HepG2 cells in part by suppressing expression of the molecules associated with hepatic gluconeogenesis.

### Effect of AWE on the expression of glycogen synthesis-related genes

**Fig. 5:**
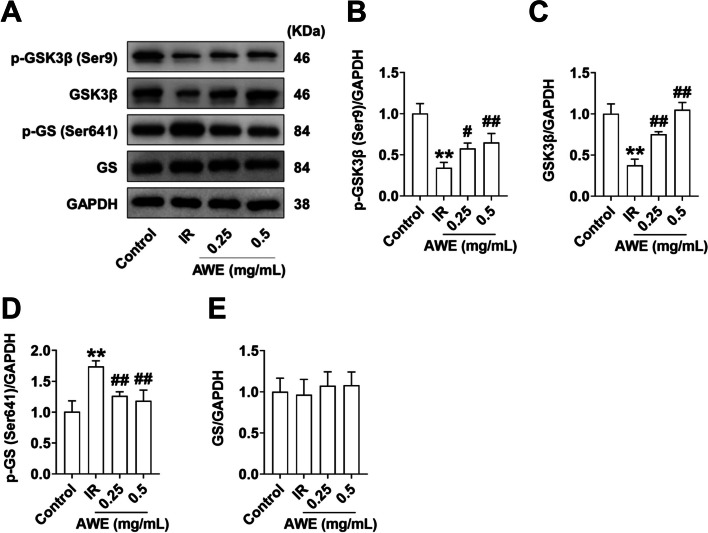
AWE enhanced the expression of key molecules involved in glycogenesis in IR HepG2 cells. HepG2 cells were exposed to 0.2 mM PA in the absence or presence of 0.25 mg/mL and 0.5 mg/mL AWE for 24 h and then incubated with 100 nM insulin for 30 min. **A** Representative Western blot images of GSK3β (Ser9), GSK3β, p-GS (Ser641), GS, and GAPDH. **B**-**E** Quantitative analysis of GSK3β (Ser9) (**B**), GSK3β (**C**), p-GS (Ser641) (**D**), GS (**E**). GAPDH is used as a loading control for protein normalization. The data are expressed as mean ± SD. *n* = 3 *per* group. ^**^P < 0.01 *vs.* Control group. ^#^*P* < 0.05, ^##^*P* < 0.01 *vs.* IR group. The experiment was repeated three times

As shown in Fig. 5A-E, there was a decrease in p-GSK3β (Ser9) (Fig. 5B) and total GSK3β (Fig. 5C) protein levels but an increase in p-GS (Ser641) (Fig. 5D) protein levels in the IR group compared to the control group (*P* < 0.01), consistent with decreased cellular glycogen content in the IR group (Fig. 2E). However, AWE significantly upregulated p-GSK3β (Ser9) protein expression (*P* < 0.01) and downregulated p-GS (Ser641) protein expression (*P* < 0.01), consistent with the increased cellular glycogen content in AWE-treated cells (Fig. 2E) with no change in the protein content of GS in these groups. These results suggest that AWE may also improve IR by promoting glycogen synthesis through regulation of GSK3β/GS signaling.

### Effect of AWE on the expression of ER stress-related genes

**Fig. 6:**
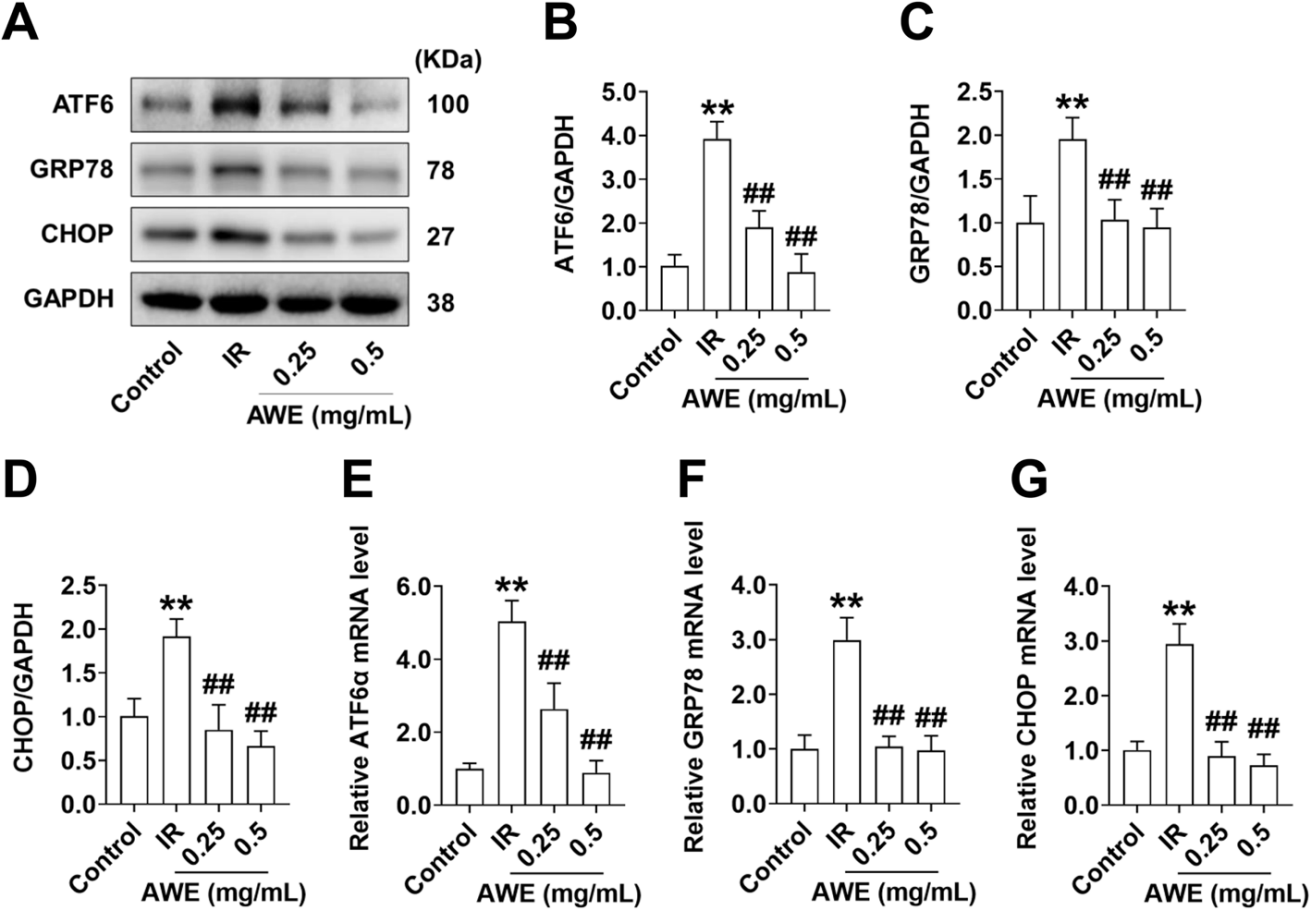
AWE suppressed the expression of ER stress sensors in IR HepG2 cells. HepG2 cells were exposed to 0.2 mM PA in the absence or presence of 0.25 mg/mL and 0.5 mg/mL AWE for 24 h and then incubated with 100 nM insulin for 30 min. **A** Representative Western blot images of ATF6, GRP78, CHOP, and GAPDH. **B**-**D** Quantitative analysis of ATF6 (**B**), GRP78 (**C**), CHOP (**D**). GAPDH is used as a loading control for protein normalization. **E**–**G** Relative mRNA expression of ATF6 (**E**), GRP78 (**F**), and CHOP (**G**) was quantified by qRT-PCR. mRNA levels of target genes were normalized to GAPDH. The data are expressed as mean ± SD. *n* = (3—6) *per* group. ^**^P < 0.01 *vs.* Control group. ^##^*P* < 0.01 *vs.* IR group. The experiment was repeated three times

To investigate whether the anti-IR effect of AWE is related to the inhibition of PA-induced ER stress, we compared the expression levels of key ER stress-sensing proteins after AWE treatment. PA induced a significant increase in both mRNA and protein levels of activating transcription factor 6 (ATF6) (Fig. 6A, B, and E), G protein-coupled receptor 78 (GRP78) (Fig. 6C, F), and the C/EBP homologous protein (CHOP) (Fig. 6D, G) (*P* < 0.01). However, AWE treatment significantly decreased the mRNA and protein expression of these ER stress sensors (*P* < 0.01). This suggests that a possible explanation for the anti-IR effects of AWE in HepG2 cells could be alleviation of PA-induced ER stress.

## Discussion

Artichoke (Cynarascolymus L.), a traditional herbal medicine known for its liver protection, has been shown to possess multiple pharmacological functions including hypolipidemic, antidiabetic, antioxidant, anti-inflammatory and anticancer effects [19]. Studies have shown that artichokes contain high levels of chlorogenic acid, cynarin, flavonoids, and other active compounds [26, 27].Among these, chlorogenic acid has been reported to improve glucose homeostasis by increasing glucose tolerance and insulin sensitivity [28] and improve high-fat diet-induced hepatic steatosis and IR in mice fed a high-fat diet [29], and flavonoid supplements improve IR in overweight and obese participants [30]. Our previous study has consistently demonstrated that artichoke water extract may attenuate IR by activating PI3K/Akt signaling in the liver of rats with nonalcoholic fatty liver disease [22]. In this study, our results revealed that AWE has an inhibitory effect on PA-induced IR in HepG2 cells by regulating glucose metabolism involved in IRS1/PI3K/Akt pathway by reducing gluconeogenesis and increasing glycogenesis, which may be due to inhibition of PA-induced ER stress.

PA, the most abundant saturated fatty acids in circulation, plays a critical role in the development of metabolic disease including IR, metabolic syndrome and T2DM [31]. Numerous studies have shown that PA can cause lipoapoptosis and IR, which are characterized by defects in glucose uptake and consumption, increased gluconeogenesis, and decreased glycogen synthesis in insulin-sensitive cells, including hepatocytes, myocytes, and adipocytes [32]. Consistent with this, our results showed that cell viability and cellular glucose consumption were reduced in a dose-dependent manner after HepG2 cells were exposed to PA. Due to the strong cytotoxic effect of high PA concentrations, 0.2 mM PA was chosen for the establishment of the IR model in HepG2 cells in the present study. Accordingly, reduced glucose uptake and reduced cellular glycogen content but increased gluconeogenesis was detected in PA-induced IR-HepG2 cells, suggesting that the IR model was successfully constructed in HepG2 cells. Metformin, the most widely used clinical insulin-sensitizing agent, improves blood glucose control through several mechanism, including suppression of hepatic glucose production, promotion of hepatic glycogen synthesis, and increasing glucose uptake and glycolysis [33]. IR-mediated dysregulated glucose metabolism was reversed by AWE treatment, including an increase in cell viability, glucose uptake and consumption, increased cellular glycogen content, and a reduction in gluconeogenesis, which is similar to the effect of metformin. These results suggest that AWE has the potential to alleviate PA-induced lipotoxicity and IR in HepG2 cells.

IRS1, a key adapter in insulin-signaling, transmits signals from insulin receptor to the PI3K/Akt pathway [2]. Previous reports showed that PA induces IR in human HepG2 cells by enhancing proteasomal degradation of key insulin signaling molecules [34]. Consistent with this, the results showed that total protein levels of key insulin signaling molecules including IRS1, PI3K p100α, Akt, and phosphorylation of IRS-1 (Tyr612) and Akt (S473) were downregulated in IR HepG2 cells. However, AWE treatment dose-dependently increased total protein levels and phosphorylation of these molecules without affecting mRNA levels, suggesting that the inhibitory effect of AWE on the PA induced degradation of key molecules involved in IRS1/PI3K/Akt signaling may be related to post-translational modifications but not to transcriptional regulation. In addition, GLUT2, the main glucose transporter of hepatocytes, whose expression is regulated by the AKT [11]. The results showed that the PA mediated downregulation of GLUT2 was reversed by AWE treatment.

FoxO1, a transcription factor of the Akt downstream target, plays an important role in insulin-mediated glucose metabolism by promoting gluconeogenesis through transcriptional activation of PEPCK and G6Pase [35]. Our data showed that in IR-HepG2 cells, a decrease in FoxO1 phosphorylation and total protein levels and an increase in PEPCK and G6Pase mRNA and protein levels were observed. Consistent with the inhibitory effect of AWE on glucose production, AWE treatment dose-dependently increased p-FoxO1(Ser 256) and FoxO1 protein expression and downregulated PEPCK and G6Pase mRNA and protein levels, suggesting that AWE might alleviate IR by inhibiting gluconeogenesis by suppressing hepatic gluconeogenic gene expression.

Insulin-mediated activation of the PI3K/Akt/GSK3β signaling pathway plays a critical role in glucose metabolism in liver and muscle cells by increasing GSK3β phosphorylation and thereby activating GS, promoting the conversion of glucose into glycogen [12]. Consistent with decreased cellular glycogen content, decreased protein levels of GSK3β (Ser9) and GSK3β but increased protein levels of p-GS (Ser641) were observed in IR-HepG2 cells. Interestingly, AWE treatment increased p-GSK3β (Ser9) and GSK3β protein levels, but decreased p-GS (Ser641) protein levels in IR-HepG2 cells, which coincided with the increased cellular glycogen content in AWE-treated cells. This suggests that AWE may also improve IR by promoting glycogen synthesis through regulation of GSK3β/GS signaling.

PA induced toxicity and IR could be mediated by ER stress, which triggers activation of the UPR and ER-associated degradation pathway by directing the ubiquitin-mediated degradation of a variety of ER-associated misfolded and normal proteins [36, 37]. Previous reports showed that PA induces IR by enhancing ubiquitination and proteasomal degradation of key insulin signaling molecules [34]. Accordingly, our results showed that total protein levels of IRS-1, PI3K(p100α), and Akt were significantly decreased in PA-treated HepG2 cells, while AWE suppressed PA-mediated degradation of these proteins. Furthermore, we found that the PA-induced upregulation of the major ER stress proteins ATF6, GRP78, and CHOP could be inhibited by AWE, suggesting that the anti-IR action of AWE could be due to inhibition of PA-mediated ER stress in HepG2 cells.

## Conclusions

This study revealed that AWE has an inhibitory impact on PA-induced IR in HepG2 cells by regulating glucose metabolism involved in IRS1/PI3K/AKT/FoxO1 and GSK-3β signaling pathway by reducing gluconeogenesis and increasing glycogenesis. These results provide a pharmacological basis for the further application of AWE in the treatment of IR-associated metabolic diseases.

### Supplementary Information

**Additional file 1. **

## Data Availability

All data generated or analysed during this study are included in this published article.

## Abbreviations

AWE: Artichoke water extract
PA: Palmitate
IR: Insulin resistance
T2DM: Type 2 diabetes mellitus
IRS1: Insulin receptor substrate 1
PI3K: Phosphatidylinositol-4,5-bisphosphate 3-kinase catalytic
Akt: Serine/threonine kinase 1
GSK3β: Glycogen synthase kinase 3 beta
FoxO1: Forkhead box transcription factor O1
ER stress: Endoplasmic reticulum stress
UPR: Unfolded protein response
