# Supplementary material for: Artichoke (Cynara scolymus L.) water extract alleviates palmitate-induced insulin resistance in HepG2 hepatocytes via the activation of IRS1/PI3K/AKT/FoxO1 and GSK-3β signaling pathway
Source: BMC Complement Med Ther. 2023 Dec 15;23:460. doi: 10.1186/s12906-023-04275-3 (PMC10722847; doi:10.1186/s12906-023-04275-3)

Artichoke (*Cynara scolymus L.*) water extract alleviates palmitate-induced insulin resistance in HepG2 hepatocytes via the activation of IRS1/PI3K/AKT/FoxO1 and GSK-3β signaling pathway

Aihua Deng[1], Yun Wang[1], Kerui Huang[1], Peng Xie[1], Ping Mo[1], Fengying Liu[1], Jun Chen[2], Kaiyi Chen[2], Yun Wang[3*], Bing Xiao[4*]

Figure 3A

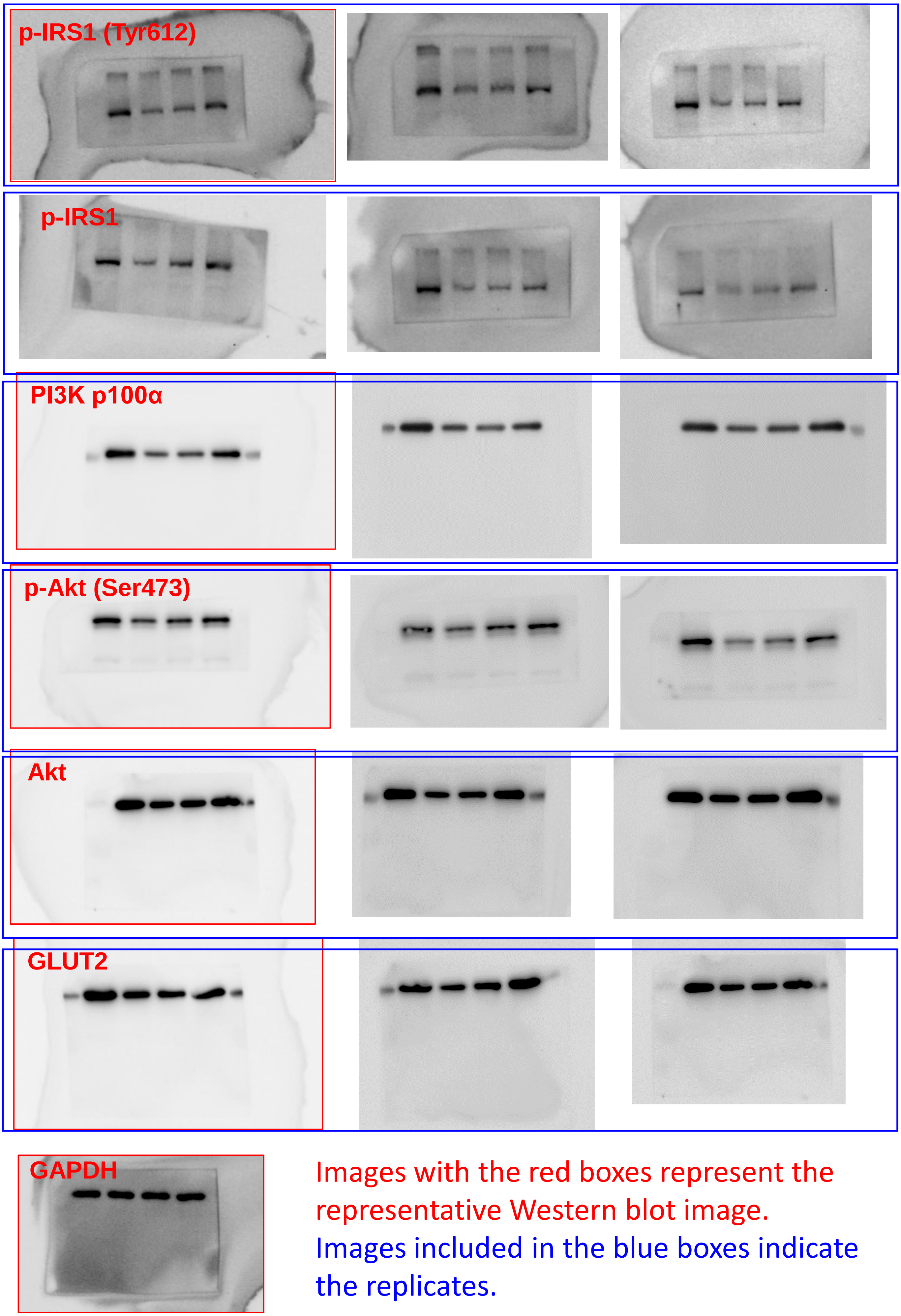

Images with the red boxes represent the representative Western blot image.

Images included in the blue boxes indicate the replicates.

Figure 4A

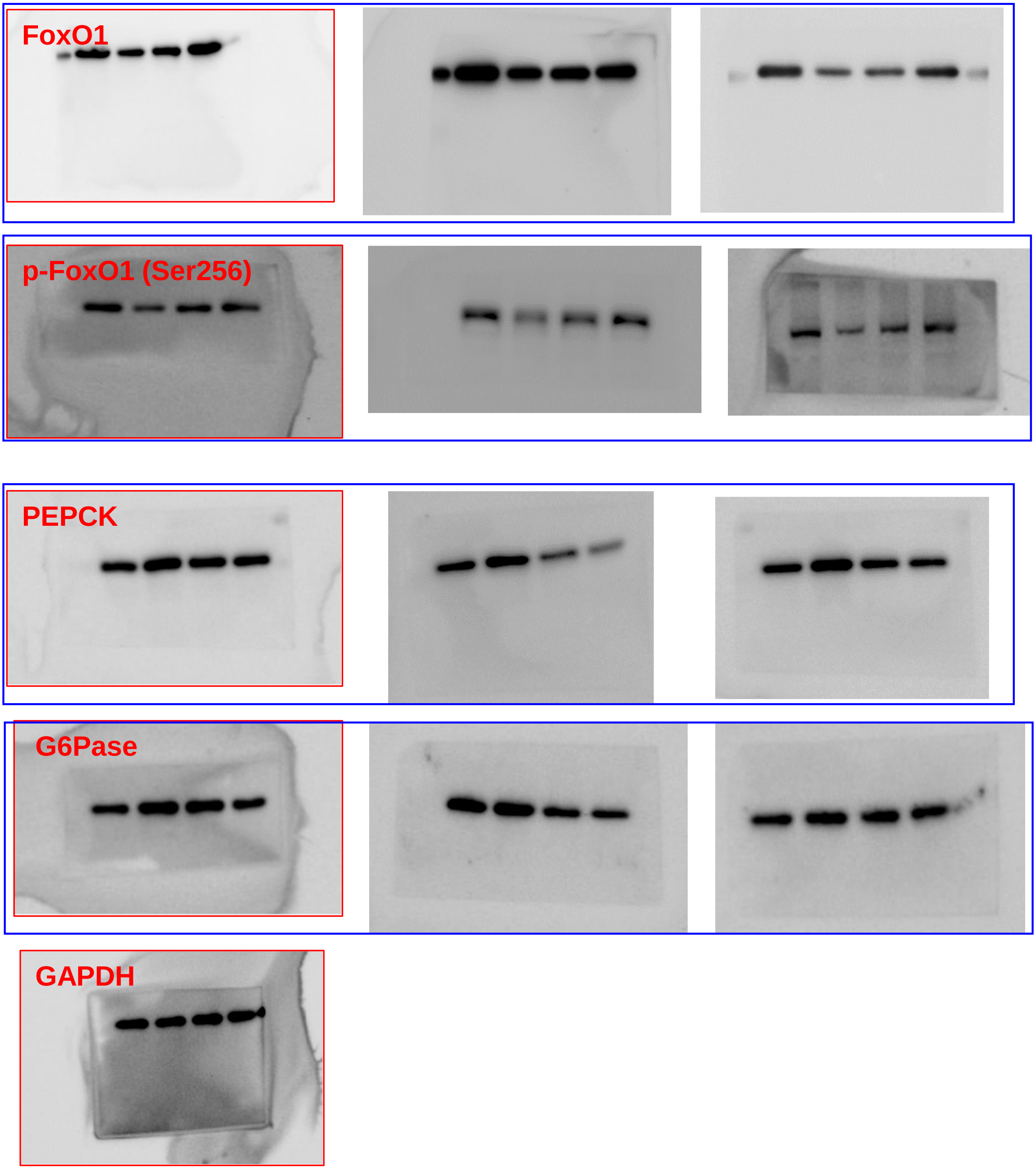

Figure 5A

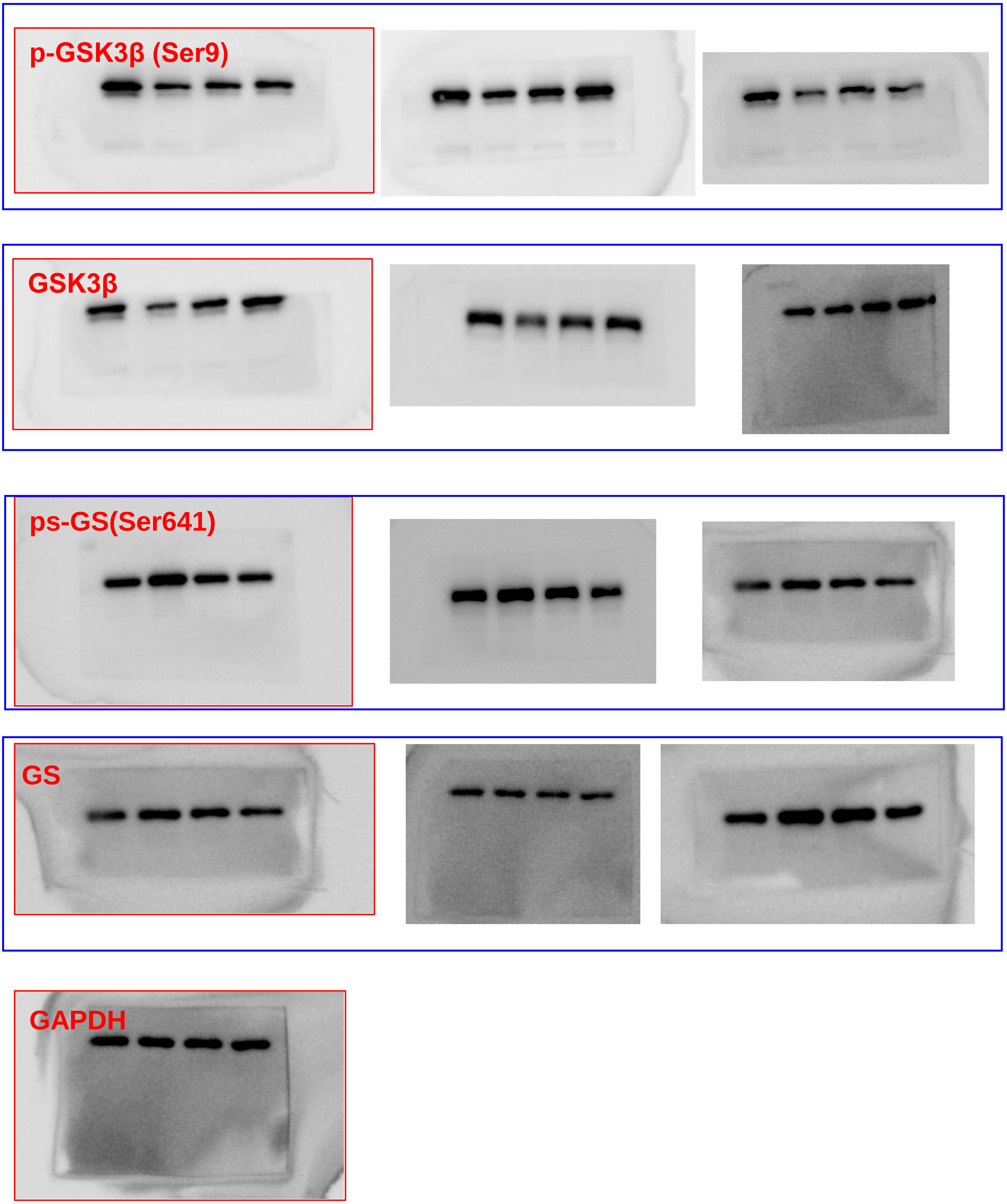

Figure 6A

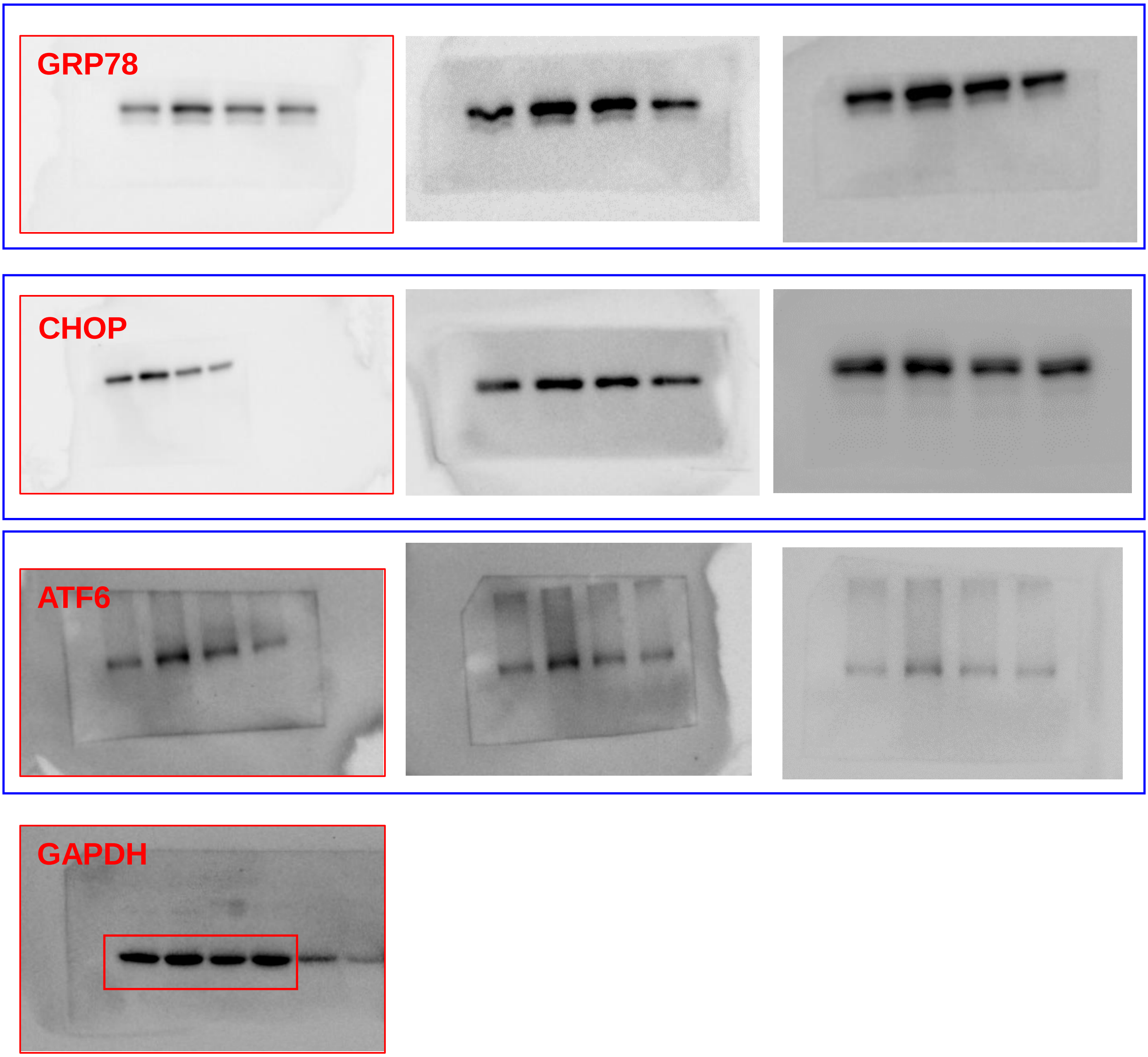

Supplement: Supplementary file 1 — Additional file 1. [file 12906_2023_4275_MOESM1_ESM.pdf]
